# Uptake of retroviral pre-exposure prophylaxis and its associated factors among female sex workers, Northwest Ethiopia

**DOI:** 10.1186/s12981-023-00573-5

**Published:** 2023-11-05

**Authors:** Belayneh Fentahun Shibesh, Aragaw Bitew Admas, Amarech Wondie Lake, Samuel Befekadu Getu, Daniel Tarekegn Worede

**Affiliations:** 1 MPH in Epidemiology, HIV/AIDS Prevention, Treatment, and Care, Amhara Regional State Health Bureau, Bahir Dar, Ethiopia; 2 MPH in Reproductive Health, HIV/AIDS prevention Treatment and Care, Amhara Regional State Health Bureau, Bahir Dar, Ethiopia; 3 HIV/AIDS prevention Treatment and Care, MPH, Amhara Regional State Health Bureau, Bahir Dar, Ethiopia; 4 MPH in Public Health in Disaster, Infection disease control, Södertärn University, Huddinge, Sweden; 5 MPH in Epidemiology, Department of Public Health, College of Medicine and Health Science, Debre Markos University, Bahir Dar, Ethiopia

**Keywords:** Pre-exposure prophylaxis, Female sex worker, Associated factors, Bahir Dar, Ethiopia

## Abstract

**Background:**

Pre-exposure prophylaxis is the use of antiretroviral medications by HIV-negative individuals to prevent infection before exposure. Ethiopia has made progress in reducing new HIV infections, but the burden remains high with ongoing challenges in prevention uptake. This study examined the utilization and factors associated with pre-exposure prophylaxis among female sex workers.

**Methods:**

A community-based cross-sectional study design was conducted in Bahir Dar city administration among female sexual workers in 2022. The results were collected using a pre-tested and structured questionnaire. Epi data for data entry and social package for social science for analysis were used.

**Result:**

Overall, 15.9% (CI: 12.0-21.1) of female sexual workers received pre-exposure prophylaxis. Parents’ living condition (only father alive [AOR = 0.23, 95% CI, 0.02–0.64], only mother alive [AOR = 0.31, 95% CI, 0.02–0.74]), marital status being single (AOR = 0.27, 95% CI, 0.06–0.94), having history of STI (AOR = 2.82, 95% CI, 1.60–4.77) were associated with pre-exposure prophylaxis uptake.

**Conclusion:**

This study showed low pre-exposure prophylaxis uptake. The study identified a history of sexually transmitted infections, marital status, and parent living conditions as significant factors. To increase pre-exposure prophylaxis uptake and reduce HIV incidence, an awareness campaign, tailored support, targeted interventions, and addressing concerns of high-risk groups are needed.

## Background

Pre-exposure prophylaxis (PrEP) is a crucial strategy in the fight against HIV transmission. It is the use of antiretroviral medications by HIV-negative individuals before potential HIV exposure. Oral PrEP, predominantly containing Tenofovir (300 mg TDF), is highly effective and a cornerstone of combination HIV prevention strategies [1, 2].

Health ministries and partners are working towards controlling the HIV epidemic and meeting the UNAIDS targets of 95-95-95, with the ultimate goal of eradicating HIV transmission by 2030. The World Health Organization emphasizes the importance of PrEP for high-risk individuals as part of HIV prevention [3].

HIV In Ethiopia is a low-intensity and mixed-type epidemic with geographic variation and population disparities. Female sex workers (FSWs) are at a 21-fold higher risk of getting HIV compared to the general population, emphasizing the need for targeted interventions [1, 3]. The HIV prevalence among adults aged 15–49 is 0.96%, 2.9% in urban vs. 0.4% in rural areas [4].

FSWs are at higher risk of HIV due to their work environment, poverty, stigma, and discrimination. Risky behaviors like having multiple sexual partners, inconsistent condom use, and drug use also increase there risk [5–7]. FSWs in Ethiopia work in hotels, bars, and streets but aren’t formally organized, making them more vulnerable to HIV infection [8].

HIV prevalence among FSW in Ethiopia was 23%, varying from 14% in Hawassa to 32% in Mekele (2013–2014) [9]. A recent Hawasa study found an 18.7% prevalence [8]. A 2022 study showed a pooled prevalence of 18.7%, highest in Bahir Dar (28.2%) and lowest in Shashemene (14.0%) [10].

Ethiopia is battling the high prevalence of HIV Infection with a combination prevention approach. Ethiopian Ministry of Health has scaled up services, including PrEP, for FSWs to reduce infection risk. However, research on PrEP uptake among FSWs in Ethiopia is scarce, hindering targeted interventions. Understanding uptake is crucial to allocate resources effectively and eliminate HIV transmission by 2030. This study assessed uptake and influencing factors among FSWs.

## Methods and materials

A community-based cross-sectional study was conducted in Bahir Dar among female sexual workers from January 1–30, 2022. The total population of Bahir Dar is about 339,683. Bahir Dar City is divided into 21 Kebeles. The city has about 4062 bars-based, home-based, and street-based FSWs.

This study included all PrEP-eligible FSWs living in Bahir Dar City for more than six months, excluding those seriously ill. The outcome variable was PrEP uptake, measured by whether eligible clients had collected the medication at least once [1]. This study defined an FSW worker as a “chewer” if she has used chewing Khat in the last month. The FSW was considered a “chewer” if she used chewing Khat and a “drinker” if she consumed alcoholic beverages in the past month [11].

We determined the sample size of 375 using EPI info software, assuming a 95% confidence level, a 5% margin of error, a 36.6% [12] proportion of PrEP, and a 5% non-response rate. Participants were randomly selected from the FSW list provided by the Bahir Dar City Health Administration . Data were collected using a structured questionnaire prepared by selecting, modifying, and adapting relevant standard evaluation tools. Two BSC nurses’ supervisors and ten nurses were required to facilitate data collection. To maintain data quality, a pretest was conducted with 10% of the participants, training for data collectors and supervisors was given for a day, and the English version of the questionnaire was translated to Amharic (local language) and then back to English to ensure consistency. The supervisor and principal investigator oversaw the entire process.

Data were entered into Epi-data v3.1 and exported to SPSS v23 for analysis. Bi-variable logistic regression was used to identify candidate variables (P < 0.25) for multivariable analysis. Multicollinearity diagnostic test was done using VIF, tolerance & standard error. Variables with P-values < 0.05 at multivariable analysis were considered as significant for PrEP uptake. The strength of theassociation was measured by adjusted odd ratio (AOR). The goodness-of-fit statistic test, Hosmer-Lemshow, was performed.

## Result

### Socio-demographic characteristics

Of 334 FSW participants, 37 (11.1%) FSWs were in a relationship with a partner called “Baluka” who is considered a husband to FSW and had the right to engage without payment and serve as guardian for FSW. The mean average age of participants were 25.6 ± 5.56 standard deviation. The response rate was 89.1% (Table 1).

### Knowledge and attitude-related factors

In this study, 27.3% (91 people) knew about PrEP. Among them, 26.4% doubted its effectiveness, while 37.4% believed it could encourage risky sexual behavior. 17.6% thought that taking PrEP would replace the need for condoms (Table 1).

**Table 1:** Socio-demographic, Knowledge, attitude, Health Facility, and Behavioral Characteristics of FSW in Bahir Dar town, Northwest, Ethiopia, 2022

Variables	Category	Numbers	Percent (%)
Educational states	Unable to read & write	6	1.8
	Able to read and write	20	6
	Grade1-8	126	37.7
	Grade 9–12	179	53.6
	Certificate and above	3	0.9
Marital status	Single	228	68.3
	Divorced /widowed	69	20.3
	With Baluka	37	11.1
Parent living condition	Divorced	34	10.2
	Both dead	32	9.6
	Only father alive	90	26.9
	Only mother alive	121	36.2
	Both alive in marriage	57	17.1
Previous Residence	Urban	70	21
	Rural	264	79
Means of information (n = 91)	Health care providers	46	49.8
	Media	4	5.2
	Friends	41	45
PrEP provided for all eligible clients (n = 91)	Yes	31	34.1
Have information and take PrEP (n = 91)	Yes	53	58.3
PrEP Prevents STI (n = 53)	Yes	12	23.5
Easy medication access from facilities (n = 53)	Yes	32	61.5
Comfortable with treatment schedule (n = 53)	Yes	39	75
Positive feeling about facility service (n = 53)	Yes	39	75

### Health facility and behavioral characteristics

Among participants, 53(15.9%) received PrEP for HIV prevention, with 58.5% receiving the service recently and 41.5% had received in the past. Of those not taking PrEP (281), 86.5% lacked information, 7.1% feared stigma, 3.2% lacked trust, 1.8% had transportation issues, and 1.4% worried about drug side effects. Additionally, among all participants, 20.1% smoked cigarettes, 39.9% chewed Khat, 88% drank alcohol, 82% consistently used condoms, and 44.3% reported condom breakage (Table 1).

### 5.5. Factors associated with PrEP uptake

The variables found to be significant in bi-variable analysis did not remain significant at multivariable analysis. Only Marital status, parents’ living situation, and history of STI were significantly associated with PrEP uptake in both bi-variable and multivariable logistic regression analysis.

FSW with a history of STIs were 2.8 times more likely to take PrEP. Single FSW were 73% less likely to take PrEP than those living with their “Baluka”. FSWs with only one living parent were less likely to take PrEP, 77% less likely when only their fathers were alive, and 69% less likely when only their mothers were alive than those whose both parents were alive (Table 2).

**Table 2:** Factors associated with PrEP uptake among FSW in Bahir Dar City, Northwest Ethiopia, 2022 (N = 334)

Variables		PrEP uptake		COR (95% CI)	AOR (95% CI)	P-value
		Yes	No			
Marital status	Single	30	198	0.36 (0.16–0.80)	0.27 (0.06–0.94)	0.04*
	Divorced/widowed	12	57	0.58 (0.19–1.27)	0.63 (0.075–2.94)	
	With Baluka	11	26	1	1	
Parent living condition	Divorced	5	29	0.41 (0.13–1.22)	0.51 (0.08–9.32)	
	Both dead	7	25	0.66 (0.24–1.81)	0.44 (0.27–4.05)	
	Only father alive	9	81	0.26 (0.11–0.64)	0.23 (0.02–0.64)	0.003*
	Only mother alive	15	106	0.33 (0.15–0.73)	0.31 (0.02–0.74)	0.007*
	Both alive in marriage	17	40	1	1	
History of STI	Yes	16	81	1.07 (0.56–2.03)	2.82 (1.60–4.77)	0.000*
	No	37	200	1	1	
Alcohol Drinking	Yes	48	246	1.37 (0.51–3.66)	0.478 (0.54–1.10)	
	No	5	35	1	1	
HIV perceived risk	Yes	24	144	0.51 (0.24–1.13)	1.92 (0.81–4.45)	
	No	17	100	0.52 (0.23–1.20)	2.05 (0.847–4.99)	
	Not sure	12	37	1	1	

Key- * =Significant with P < 0.05, 1 = reference category

## Discussion

Ethiopia has made significant efforts to reduce the impact of HIV, leading to substantial progress in reducing the number of new HIV infections. However, despite achievements, the burden of HIV remains high, and there is an ongoing challenge in the uptake of HIV preventive measures. This study tried to find the uptake of PrEP and its associated factors among FSWs.

According to the study, only 15.9% (CI: 12.0-21.1) of FSW had received PrEP. The result is in agreement with previous studies of Atlanta (11.9%) [13] and Kenya Kenya (21.7%) [14], but lower compared to studies carried out in South Africa (36.6%) [12], Brazil (60.9%) [15], and Uganda (92.2%) [16]. The difference in PrEP uptake among FSWs could be due to various reasons. These include the local epidemic intensity, specific demographic and behavioral factors, awareness of PrEP, availability and accessibility of PrEP medication, attitude of healthcare providers and facilities, and general attitude towards PrEP within the population. Factors such as stigma, misconception, and cultural beliefs can also influence whether FSWs are willing to use PrEP.

In this study, lack of information was the main barrier to accessing PrEP services, while studies in Kenya and Uganda identified side effects and stigma [14, 16]. Possible reasons for this difference could include variations in program implementation and promotion. There may be shortcomings in public health campaigns or healthcare provider communication regarding PrEP benefits and safety. In Kenya and Uganda, side effects and stigma were highlighted, indicating better awareness about PrE. Cultural factors and resource availability may also differ among countries, contributing to variations in perceived barriers.

The study found that PrEP uptake was significantly associated with a history of STIs, marital status, and parent living conditions among FSWs. Those with a history of STIs had higher odds of PrEP uptake and might be more aware of PrEP as a prevention measure. This finding was consistent with a previous study [17, 18]. This could be due to frequent interaction with healthcare providers or HIV/STI prevention services. FSWs with STIs might also have established connections with healthcare services, making it easier to access PrEP and receive education and counseling about HIV prevention methods. Those with a history of STIs may also perceive themselves at higher risk of HIV and be motivated to seek out PrEP.

This study found that single FSWs were less likely to receive PrEP than those in committed relationships (“Baluka”) which is supported by previous study [18]. This might be because single FSWs lack social support to access PrEP, while those in committed relationships may be encouraged by their partners. Additionally, FSWs may face financial constraints and perceive a higher risk of contracting HIV, making PrEP a necessary preventive measure for both partners and FSWs.

In the current study, parents’ living conditions contributed to the uptake of PrEP. FSWs with only one living parent are less likely to use PrEP than those with both parents alive and in a marital relationship. This could be due to additional psychological stressors or challenges faced by those who have experienced the loss of a parent. Additionally, individuals with a stable family support system, including emotional, financial, and healthcare support, may be more encouraged and facilitated to use preventive measures like PrEP.

## Conclusions

The study founds low uptake of PrEP. STI history, being single, and parental living situations associated with its use. Lack of information is the main reason for not using PrEP. To increase uptake, a multifaceted approach is needed: awareness campaigns, tailored support, targeted interventions, and addressing concerns of at-risk populations.

### Recommendations

Create educational content to raise PrEP awareness among at-risk individuals and healthcare providers. Involve community leaders and conduct qualitative research to better understand the motivations for and barriers to PrEP use.

## Data Availability

The data from this study can be obtained from the corresponding author upon reasonable request.

## Abbreviations

ART: Anti-Retroviral Therapy
CI: Confidence Interval
FSW: Female Sexual Workers
AOR: Adjusted Odd Ratio
OR: Odds Ratio
PrEP: Preexposure Prophylaxis
STI: Sexually transmitted infection
